# A mathematical model for the investigation of combined treatment of radiopharmaceutical therapy and PARP inhibitors

**DOI:** 10.1007/s00259-025-07144-y

**Published:** 2025-02-20

**Authors:** Marc Ryhiner, Yangmeihui Song, Jimin Hong, Carlos Vinícius Gomes Ferreira, Axel Rominger, Susanne Kossatz, Gerhard Glatting, Wolfgang Weber, Kuangyu Shi

**Affiliations:** 1 Department of Nuclear Medicine, Inselspital, University of Bern, Bern, Switzerland; 2 Department of Nuclear Medicine, TUM University Hospital, School of Medicine, Technical University of Munich, Munich, Germany; 3 Medical Radiation Physics, Department of Nuclear Medicine, Ulm University, Ulm, Germany; 4 Department of Nuclear Medicine, Union Hospital, Tongji Medical College, Huazhong University of Science and Technology, No. 1277 Jiefang Ave, Hubei Province 430022 Wuhan, China

**Keywords:** Radiopharmaceutical therapy, Digital twin, PARP inhibitor, Homologous recombination deficiency, Therapy simulation, Therapy optimization

## Abstract

**Background:**

Although the combined treatment with radiopharmaceutical therapy (RPT) and poly (ADP-ribose) polymerase inhibitors (PARPi) shows promise, a critical challenge remains in the limited quantitative understanding needed to optimize treatment protocols. This study introduces a mathematical model that quantitatively represents homologous recombination deficiency (HRD) and facilitates patient-specific customization of therapeutic schedules.

**Methods:**

The model predicts therapeutic outcomes based on the absorbed dose by DNA and the resulting radiobiological responses, with DNA double-strand breaks (DSBs) being the critical determinant of cancer cell fate. The effect of PARPi is modeled by the accelerated conversion of single-strand breaks (SSBs) to DSBs due to PARP-trapping in the S phase, while HRD is represented by defects in DSB repair in replicated DNA. In vitro experiments are used to calibrate the model parameters and validate the model. *In silico* tests are designed to extensively investigate various combination protocols including the LuPARP trial.

**Results:**

Model calibration was performed using data from the treatment of NCI-H69 cells with [^177^Lu]Lu-DOTA-TOC and PARPi. Previously published in vivo studies were integrated into the presented model. Model validation using in vitro data showed deviations within the experimental error margins, with average deviations of 5.3 ± 3.2% without PARPi, 6.1 ± 4.4% with Olaparib, and 12 ± 18% with Rucaparib. Rucaparib radiosensitization reduces number of tumor cells during lutetium therapy by 99.2% and 99.99% (HRD). The highest radiosensitizing effect in vivo and in vitro was observed with Talazoparib (IC50: 4.8 nM), followed by Rucaparib (IC50: 1.4 µM). The model predicts relative tumor shrinkage after 14 days of combination treatment with Olaparib (250 mg) based on patient body weight (e.g. 60 kg: 99.6%; 90 kg: 98.0%).

**Conclusion:**

Results demonstrate the potential of this computational model as a step toward the development of the digital twin for systematic exploration and optimization of clinical protocols.

**Supplementary Information:**

The online version contains supplementary material available at 10.1007/s00259-025-07144-y.

## Background

Radiopharmaceutical therapy (RPT) is a cancer treatment designed to deliver radiation absorbed doses to cancer cells via cancer-targeting radiopharmaceuticals. To enhance the therapeutic response, lutetium combination therapies have been explored, including alpha therapy and immune checkpoint therapy (ICT) [1, 2]. Additionally, combination treatments involving poly (ADP-ribose) polymerase inhibitors (PARPi) have been proposed to improve the anti-tumor efficacy of lutetium therapy [3].

PARPi trap DNA repair intermediates at DNA single-strand break (SSB) sites, forming PARP traps [4]. During DNA replication, these PARP traps are converted into DNA double-strand breaks (DSBs), which are the primary cause of radiation-induced cell death [5]. Cancers with mutations in the homologous recombination (HR) pathway, such as defective BRCA1/2 genes, show promising responses to PARPi treatment [6]. Both PARPi monotherapy and RPT have demonstrated effectiveness in clinical trials for HR-deficient (HRD) cancers [7, 8]. Following these successes, investigating PARPi radiosensitization of HRD cancers is of significant interest.

Digital twins have made substantial contributions to various oncological fields, including personalized medicine [9]. Unlike the instantaneous dose deposition in external beam radiotherapy (EBRT), dose delivery in RPT is continuous and depends on factors like tumor vascularization and ligand concentration within the tumor microenvironment. Similar to EBRT, radiobiological models are essential for personalized treatment planning in RPT, providing quantitative insights into dose-response and dose-toxicity relationships [10]. However, current radiobiological models, such as the linear quadratic (LQ) model, describing EBRT, are not directly applicable to RPT [11]. A model for predicting cancer cell survival during EBRT exists [12], but no model currently investigates the combination of RPT and PARPi therapy.

This study proposes the first computational model to facilitate the investigation of combined RPT and PARPi treatment. The model incorporates parameters such as DNA damage, damage repair systems, cell cycle heterogeneities, and DNA replication to assess cell survival. The current stage of this investigation provides RPT microdosimetry using an in vitro compartment model and includes PARPi radiosensitization and HRD. Additionally, in vivo data are integrated into the model, and clinical predictions are made.

## Methods

**Fig. 1:**
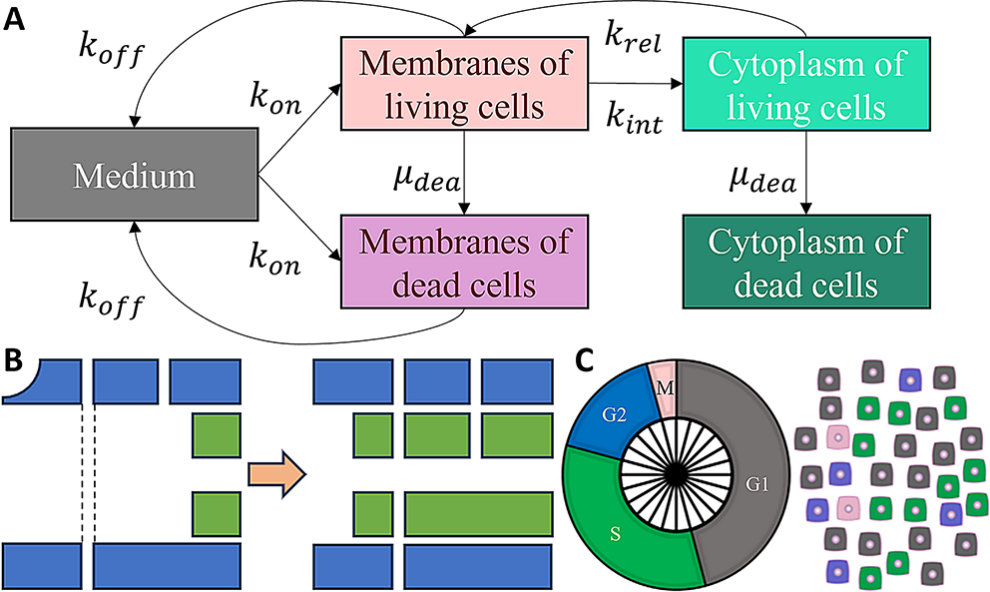
Overview of the presented RPT model. (**A**) Compartmental model depicting radiopharmaceutical transitions. (**B**) DNA replication during the S phase, with pre-existing strands shown in blue and newly synthesized strands in green. DNA strand breaks are represented by attenuated lines; single-strand breaks (SSBs) are converted to double-strand breaks (DSBs) during replication, and existing DSBs are propagated into the newly replicated DNA. (**C**) Cell cycle phase distribution, where 24 slices represent the possible cell cycle locations at the beginning of the experiment

### Cell culture

5,000 NCI-H69 suspension cells were seeded in 96-well black clear V-bottom plates containing 100 µL of 0.5% (v/v) DMSO, 24 h before adding [^177^Lu]Lu-DOTA-TOC (1–100 kBq), and Olaparib or Rucaparib (both 5 µM). The treatment exposure time was 24 h for radiopharmaceuticals and 72 h for PARPi, with washing steps involving centrifugation and resuspension. The number of surviving cells was determined using the AlamarBlue high sensitivity (HS) assay. For this, the medium was replaced 96 h after seeding with AlamarBlue HS solution, and the cells were incubated for 1–2 h at 37 °C. Subsequent fluorescence measurements were recorded directly after incubation using a Biotek Synergy HT plate reader (Excitation: 540/35, Emission: 590/20 nm). Relative number of surviving cells for each condition was calculated in relation to untreated wells. Three biological repeats with 3–6 technical replicates were recorded.

### In vitro RPT microdosimetry

Microdosimetry is evaluated using a radionuclide compartment model and the MIRD formalism (Fig. 1A) [13]. It is assumed that the radionuclide concentration in the medium ($\:{c}_{m}$) is affected by interactions with target receptors, irrespective of the cell’s viability. However, radionuclides are only able to enter and exit the cytoplasm of living cells.

1 $$\begin{aligned}{d}_{t}{c}_{m}\left(t\right)&={k}_{off}\left[{c}_{acs}\left(t\right)+{c}_{dcs}\left(t\right)\right]-{k}_{on}{c}_{m}\left(t\right)\\&\left[{R}_{alive}\left(t\right)+{R}_{dead}\left(t\right)-{c}_{acs}\left(t\right)-{c}_{dcs}\left(t\right)\right]\end{aligned}$$

where $\:{k}_{on}$ and $\:{k}_{off}$ are association and dissociation rates, respectively, and $\:t$ is time after the experiment start. $\:{R}_{alive}$ and $\:{R}_{dead}$ are receptor concentrations in living and dead cells, respectively, where $\:{R}_{alive}\left(t\right)={{R}_{cell}N}_{alive}\left(t\right)$ and $\:{R}_{dead}\left(t\right)={{R}_{cell}N}_{dead}\left(t\right)$. $\:{N}_{alive}$ and $\:{N}_{dead}$ represent the number of living and dead cells, and $\:{R}_{cell}$ is the receptor concentration contributed by a single cell.

Radionuclide concentrations on the cell surfaces of living cells ($\:{c}_{acs}$) are determined by each possible compartment change, whereby the concentration of the cell surfaces of dead cells ($\:{c}_{dcs}$) is not influenced by integration and release processes. Additionally, radionuclides bound to dying cells transition from the cell surface compartment from living cells to that of dead cells.

2 $$\begin{aligned}{d}_{t}{c}_{acs}\left(t\right)&={k}_{on}{c}_{m}\left(t\right)\left[{R}_{cell}-{c}_{acs}\left(t\right)\right]+{k}_{rel}{c}_{acy}\left(t\right)\\&-\left[{k}_{off}+{k}_{int}+{\mu\:}_{dea}\left(t\right)\right]{c}_{acs}\left(t\right)\end{aligned}$$

3 $$\begin{aligned}{d}_{t}{c}_{dcs}\left(t\right)&={k}_{on}{c}_{m}\left(t\right)\left[{R}_{death}-{c}_{dcs}\left(t\right)\right]\\&+{\mu\:}_{dea}\left(t\right){c}_{acs}\left(t\right)-{k}_{off}{c}_{dcs}\left(t\right)\end{aligned}$$

where $\:{k}_{int}$ and $\:{k}_{rel}$ are integration and release rates, respectively, and $\:{\mu\:}_{dea}$ is the cell death rate.

The radionuclide concentration within the cytoplasm of living cells ($\:{c}_{acy}$) is governed by receptor integration and cell death, while the concentration in the cytoplasm of dead cells ($\:{c}_{dcy}$) is solely influenced by cell death.

4 $$\:{d}_{t}{c}_{acy}\left(t\right)={k}_{int}{c}_{acs}\left(t\right)-\left[{k}_{rel}+{\mu\:}_{dea}\left(t\right)\right]{c}_{acy}\left(t\right)$$

5 $$\:{d}_{t}{c}_{dcy}\left(t\right)={\mu\:}_{dea}\left(t\right){c}_{acy}\left(t\right)$$

The absorbed dose ($D$) in cellular nuclei is calculated based on the time-integrated activity and the S values of the individual compartments [13]. Crossfire effects are incorporated by accounting for the intercellular medium effect.

6 $$\:{d}_{t}D\left(t\right)=\frac{A\left(t\right)\left[{S}_{m}\left[\sum\:c\left(t\right)\right]+{S}_{cs}{c}_{acs}\left(t\right)+{S}_{cy}{c}_{cy}\left(t\right)\right]}{\left[\sum\:c\left(t\right)\right]{N}_{alive}\left(t\right)}$$

where, $$\:{S}_{m}$$$$\:{S}_{cs}$$, and $$\:{S}_{cy}$$ represent the S values corresponding to the absorbed dose in the cellular nucleus from decays in the medium, on the cell surface, and in the cytoplasm, respectively. $$\:A$$ denotes the radionuclide activity in the in vitro system, where $$\:A\left(t\right)={A}_{0}{e}^{-{\mu\:}_{dec}t}$$, with $$\:{A}_{0}$$ as the initial activity and $$\:{\mu\:}_{dec}$$ the decay rate of the specific radionuclide. $$\:\sum\:c\left(t\right)$$ represents the total radionuclide concentration across all compartments. For dosimetry purposes, it is assumed that $$\:{N}_{alive}\left(t\right)\ge\:1$$, which implies that $$\:{R}_{alive}\left(t\right)\ge\:{R}_{cell}$$.

### Radiobiological response across cell phases

The number of SSBs ($\:{N}_{ssb}$) is determined by their induction due to radiation and their repair when not in S phase.

7 $$\:{d}_{t}{N}_{ssb}\left(t\right)={N}_{gen}{k}_{ssb}{d}_{t}D\left(t\right)-{\lambda\:}_{ssb}{N}_{ssb}\left(t\right)$$

where $\:{N}_{gen}$ is the number of present genomes, $\:{k}_{ssb}$ is the number of induced SSBs per radiation dose and genome, and $\:{\lambda\:}_{ssb}$ is the rate of physiological SSB repair.

According to MEDRAS, DSBs are distinguished by the kinetics with which they are repaired [12]. Excluding the S phase, the number of DSBs with fast ($\:{N}_{dsb}^{f}$) and slow ($\:{N}_{dsb}^{s}$) kinetics is influenced only by their induction and subsequent repair.

8 $$\:{d}_{t}{N}_{dsb}^{f}\left(t\right)={p}_{f}{N}_{gen}{k}_{dsb}{d}_{t}D\left(t\right)-{\lambda\:}_{dsb}^{f}{N}_{dsb}^{f}\left(t\right)$$

9 $$\:{d}_{t}{N}_{dsb}^{s}\left(t\right)={p}_{s}{{N}_{gen}k}_{dsb}{d}_{t}D\left(t\right)-{\lambda\:}_{dsb}^{s}{N}_{dsb}^{s}\left(t\right)$$

where $\:{\lambda\:}_{dsb}^{f}$ and $\:{\lambda\:}_{dsb}^{s}$ are the repair rates of fast and slow repair kinetics, respectively, and $\:{k}_{dsb}$ is the number of induced DSBs per radiation dose and genome. $\:{p}_{f}$ and $\:{p}_{s}$ are the probabilities that a DSB is repaired with fast and slow kinetics, respectively, whereby $\:{p}_{f}=(1-{p}_{c})$, where $\:{p}_{c}$ is the probability that a radiation-induced DSB is complex (cDSB). $\:{p}_{s}={p}_{c}$ throughout the cell cycle in case of full HR capability, otherwise, this equivalence holds only during the G1 phase. In case of HRD during G2 and M phase,$\:\:{p}_{s}={p}_{c}{p}_{HR}$, where $\:{p}_{HR}$ is the probability that a HR event succeeds.

The number of DSBs repaired with very slow kinetics ($\:{N}_{dsb}^{m}$) in the G2 and M phase is determined by their induction and repair.

10 $$\:{d}_{t}{N}_{dsb}^{m}\left(t\right)={p}_{m}{N}_{gen}{k}_{dsb}{d}_{t}D\left(t\right)-{\lambda\:}_{dsb}^{m}{N}_{dsb}^{m}\left(t\right)$$

where $\:{\lambda\:}_{dsb}^{m}$ is the repair rate of the microhomology-mediated end joining (MMEJ) pathway, and $\:{p}_{m}$ is the probability that a DSB is repaired via MMEJ, where $\:{p}_{m}={p}_{c}(1-{p}_{HR})$.

MEDRAS assesses cancer cell survival ($\:S$) due to genomic loss, apoptosis during the G1 phase, and mitotic catastrophe during the G2 and M phases [12]. These three survival terms and PARPi determine the overall cellular survival rate.

11 $$\begin{aligned}{d}_{t}S\left(t\right)&=-{\mu\:}_{dea}\left(t\right)S\left(t\right)\\&=\left[{\mu\:}_{loss}\left(t\right){\mu\:}_{apop}\left(t\right){\mu\:}_{mitot}\left(t\right){\mu\:}_{PARPi}-1\right]S\left(t\right)\end{aligned}$$

where $\:{\mu\:}_{loss}$ is the cell survival rate according to genomic loss, modeled as a quadratic fit with respect to $\:{N}_{dsb}\left(t\right)$, with $\:{\mu\:}_{loss}\left(t\right)=\left[a\left(t\right){N}_{dsb}\left(t\right)+b\left(t\right)\right]{N}_{dsb}\left(t\right)+1$, where $\:a$ and $\:b$ are characteristic parameters for repair kinetics distribution, and $\:a,\:b\le\:0$. $\:{N}_{dsb}$ is the number of present DSBs, whereby $\:{N}_{dsb}\left(t\right)={N}_{dsb}^{f}\left(t\right)+{N}_{dsb}^{s}\left(t\right)+{N}_{dsb}^{m}\left(t\right)$. Cell survival rates due to apoptosis and mitotic catastrophe are referred to as $\:{\mu\:}_{apop}$ and $\:{\mu\:}_{mitot}$, respectively, with $\:{\mu\:}_{apop}\:={e}^{-\psi\:{N}_{ind}\left(t\right)}$ and $\:{\mu\:}_{mitot}\:={e}^{-\phi\:{N}_{dsb}\left(t\right)}$, where $\:\psi\:$ and $\:\phi\:$ are rates for apoptosis and mitotic catastrophe, respectively. $\:{N}_{ind}$ is the number of induced DSBs within a defined time interval, where $\:{N}_{ind}\left(t\right)={k}_{dsb}{d}_{t}D\left(t\right)$. When $\:{\mu\:}_{mitot}\left(t\right)$ is assessed, $\:{N}_{dsb}\le\:20$ is assumed, representing the G2/M cell cycle check point [12]. $\:{\mu\:}_{PARPi}$ is a constant survival term representing PARPi monotherapy.

The number of living cells in the in vitro system is determined by cell death and cell duplication, while the number of dead cells is determined by cell death only.

12 $$\:{d}_{t}{N}_{alive}\left(t\right)=\left[{\mu\:}_{gr}-{\mu\:}_{dea}\left(t\right)\right]{N}_{alive}\left(t\right)$$

13 $$\:{d}_{t}{N}_{dead}\left(t\right)={\mu\:}_{dea}\left(t\right){N}_{alive}\left(t\right)$$

where $\:{\mu\:}_{gr}$ is the cell growth rate.

### S phase

DNA lesions during S phase are distinguished by their location on unreplicated (url) and replicated (rl) DNA sites. SSBs are subtracted during their replication because they are converted into DSBs (Fig. 1B).

14 $$\begin{aligned}{d}_{t}{N}_{ssb}^{url}\left(t\right)&={k}_{ssb}\left[1-{s}_{prog}\left(t\right)\right]{d}_{t}D\left(t\right)\\&-\left[{\lambda\:}_{ssb}+\frac{{\mu\:}_{inter}}{1-{s}_{prog}\left(t\right)}\right]{N}_{ssb}^{url}\left(t\right)\end{aligned}$$

15 $$\:{d}_{t}{N}_{ssb}^{rl}\left(t\right)={k}_{ssb}{s}_{prog}\left(t\right){d}_{t}D\left(t\right)-{\lambda\:}_{ssb}{N}_{ssb}^{rl}\left(t\right)$$

where $\:{s}_{prog}$ represents the proportional progression of the S phase, and $\:{\mu\:}_{inter}$ is the S phase progression per time interval, thus $\:{d}_{t}{s}_{prog}={\mu\:}_{inter}$. It is assumed that $\:{s}_{prog}\left(t\right)\ge\:{\mu\:}_{inter}-1$.

Similar to the replication of SSBs, the replication of DSBs lead to their duplication (Fig. 1B).

16 $$\begin{aligned}{d}_{t}{N}_{dsb}^{f,\:url}\left(t\right)&={k}_{dsb}\left[1-{s}_{prog}\left(t\right)\right]{d}_{t}D\left(t\right)\\&-\left[{\lambda\:}_{dsb}^{f}+\frac{{\mu\:}_{inter}}{1-{s}_{prog}\left(t\right)}\right]{N}_{dsb}^{f,\:url}\left(t\right)\end{aligned}$$

17 $$\begin{aligned}{d}_{t}{N}_{dsb}^{f,\:rl}\left(t\right)&={2k}_{dsb}{s}_{prog}\left(t\right){d}_{t}D\left(t\right)\\&+2\frac{{\mu\:}_{inter}}{1-{s}_{prog}\left(t\right)}{N}_{dsb}^{f,\:url}\left(t\right)\\&-{\lambda\:}_{dsb}^{f}{N}_{dsb}^{f,\:rl}\left(t\right)\end{aligned}$$

Transition from pre- to post-S phase repair is modeled to proceed synchronously with DNA replication due to the preferred usage of a homologous sister chromatid as template strand for the HR pathway [14].

18 $$\begin{aligned}{d}_{t}{N}_{dsb}^{s,\:url}\left(t\right)&={k}_{dsb}\left[1-{s}_{prog}\left(t\right)\right]{d}_{t}D\left(t\right)\\&-\left[{\lambda\:}_{dsb}^{s}+\frac{{\mu\:}_{inter}}{1-{s}_{prog}\left(t\right)}\right]{N}_{dsb}^{s,\:url}\left(t\right)\end{aligned}$$

19 $$\begin{aligned}{d}_{t}{N}_{dsb}^{s,\:rl}\left(t\right)&={2k}_{dsb}{s}_{prog}\left(t\right){d}_{t}D\left(t\right)\\&+\frac{{p}_{s}{\mu\:}_{inter}}{\left[{p}_{s}+{p}_{m}\right]\left[1-{s}_{prog}\left(t\right)\right]}\\&\left[2{N}_{dsb}^{s,\:url}\left(t\right)+{N}_{ssb}^{url}\left(t\right)\right]\\&-{\lambda\:}_{dsb}^{s}{N}_{dsb}^{s,\:rl}\left(t\right)\end{aligned}$$

DNA replication marks the transition from pre- to post-S phase $\:{p}_{s}$.

20 $$\begin{aligned}{d}_{t}{N}_{dsb}^{m}\left(t\right)&={2k}_{dsb}{s}_{prog}\left(t\right){d}_{t}D\left(t\right)\\&+\frac{{p}_{m}{\mu\:}_{inter}}{\left[{p}_{s}+{p}_{m}\right]\left[1-{s}_{prog}\left(t\right)\right]}\\&\left[2{N}_{dsb}^{s,\:url}\left(t\right)+{N}_{ssb}^{url}\left(t\right)\right]-{\lambda\:}_{dsb}^{m}{N}_{dsb}^{m}\left(t\right)\end{aligned}$$

For the S phase, Eq. 11 holds, with $$\:{\mu\:}_{loss}$$ modified to account for unrepaired and repaired DSBs. Therefore, $$\:{\mu\:}_{loss}\left(t\right)=\left[\left[{a}_{0}\left(t\right){N}_{dsb}^{url}\left(t\right)+{b}_{0}\left(t\right)\right]{N}_{dsb}^{url}\left(t\right)+1\right]]$$$$\left[\left[{a}_{1}\left(t\right){N}_{dsb}^{rl}\left(t\right)+{b}_{1}\left(t\right)\right]{N}_{dsb}^{rl}\left(t\right)+1\right]$$, where $$\:{N}_{dsb}^{url}$$ and$$\:\:{N}_{dsb}^{rl}$$ are the numbers of present DSBs per DNA copy on unreplicated and replicated DNA, respectively, with $$\:{N}_{dsb}^{url}\left(t\right)={N}_{dsb}^{f,\:url}\left(t\right)+{N}_{dsb}^{s,\:url}\left(t\right)$$ and $$\:{N}_{dsb}^{rl}\left(t\right)={N}_{dsb}^{f,\:rl}\left(t\right)+{N}_{dsb}^{s,\:rl}\left(t\right)+{N}_{dsb}^{m}\left(t\right)$$. $$\:{a}_{0}$$ and $$\:{b}_{0}$$ are characteristic for unreplicated DNA and $$\:{a}_{1}$$ and $$\:{b}_{1}$$ for replicated DNA.

### Parameter estimation

The parameter values were carefully optimized to maximize the physiological relevance of each parameter while minimizing overfitting. Kinetic parameters$\:\:{k}_{on}$, $\:{k}_{off}$, $\:{k}_{int}$, and $\:{k}_{rel}$ were fitted, $\:{R}_{cell}$ was calculated, dosimetry S values are simulated, and a literature value is adopted for $\:{\mu\:}_{dec}$. Considering radiobiological parameter values, SSB-related parameter values $\:{k}_{ssb}$ and $\:{\lambda\:}_{ssb}$ were adopted from the literature, except for $\:{\lambda\:}_{ssb}$ in the presence of Rucaparib. The alternative Rucaparib models assumes that PARP inhibition causes the SSB repair capability to decrease with increasing $\:{N}_{ssb}$, requiring the fitted reduction rate for SSB repair $\:{\lambda\:}_{ssb}^{red}$. The Rucaparib-influenced $\:{\lambda\:}_{ssb}$ was fitted according to experimental data.

DSB-related parameters $\:{k}_{dsb}$, $\:{\lambda\:}_{dsb}^{f}$, $\:{\lambda\:}_{dsb}^{s}$, $\:{\lambda\:}_{dsb}^{m}$, and $\:{p}_{c}$ were provided by MEDRAS [12]. Those parameters are specific for X-rays and primarily depend on linear energy transfer (LET), which is highly similar for beta particles [15]. Parameters $\:a$ and $\:b$, also reliant on this LET similarity, were calibrated using MEDRAS [12]. Survival parameter values $\:\psi\:$ and $\:\phi\:$ were adopted from MEDRAS [12], with modifications to reflect the cell cycle considerations used. Constant survival terms of $\:{\mu\:}_{PARPi}$ therapies were fitted.

Cell cycle progression rates are based on literature values assuming a general human cell phase distribution and NCI-H69 specific progression. Please find listings of used parameter values and details about the parameter $\:a$ and $\:b$ calibration process in the *Supplementary Information*.

### Cell cycle heterogeneities and result generation

Temporal cell cycle heterogeneity is represented by 24 evenly spaced experimental starting points across the cell cycle (Fig. 1C). Mean results of different starting points represent outcomes. Experiment starts 24 h after cell seeding. The cell cycle is divided into phases G1, S, G2, M with duration proportions of 11:8:4:1, equivalent to predomination of 1 present genome 62.5% of the time, compared to 67% assumed in MEDRAS [12]. Cell phase distributions of specific cell types may vary from assumed proportions, but the number of completed cell cycles corresponds to the individuality of NCI-H69 cells.

Initial values follow the assumption that radionuclides are added to undamaged cells 24 h after they were seeded and have grown according to the Malthusian population model and $\:{\mu\:}_{gr}$. Initial radionuclide concentration is determined by initial activity, molar activity, and system volume. It is assumed that the radionuclide removal excised the dead cells too. Remaining activity within the system is due to enduring radionuclides in and on living cells. Please find listings of initial variable values and variable value changes caused by radionuclide removal in the *Supplementary Information*. Results are generated be numerically solving the differential equation system using the LSODA method of the solve_ivp algorithm in SciPy version 1.13.0.

## Results

### RPT simulations describe experimental results and suggest PARPi effect on SSB repair kinetics

**Fig. 2:**
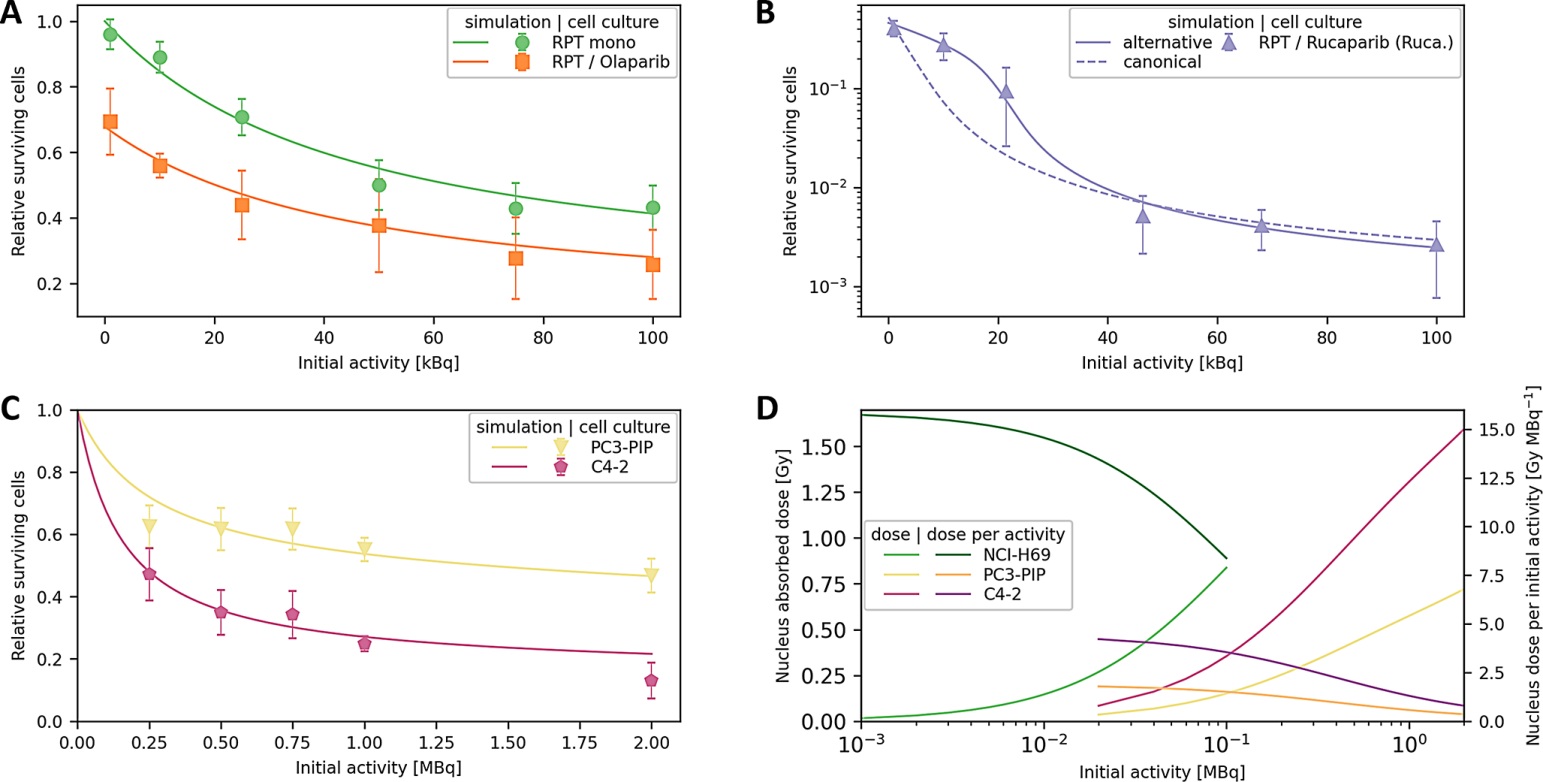
Simulated *in silico* replicates of performed in vitro experiments where NCI-H69 cells are treated with SSTR2-targeting [^177^Lu]Lu-DOTA-TOC and different PARP inhibitors [16]. Results relative to untreated cells. Error bars indicate the standard error of the mean. (**A**) Comparison on a linear scale between RPT monotherapy (mono) and Olaparib combination treatment. (**B**) Simulation on a logarithmic scale of RPT and Rucaparib combination treatment. Alternative SSB repair model treats PARP-trap clearance as a decaying function of their abundance. (**C**) Treatment of prostate cancer cell lines with [^177^Lu]Lu-PSMA-I&T. (**D**) Absorbed dose in cancer cell nuclei at the experimental endpoints

Kinetic parameters $\:{k}_{on}$, $\:{k}_{off}$, $\:{k}_{int}$, and $\:{k}_{rel}$ were calibrated using in vitro data of RPT monotherapy experiments. A temporal discrepancy between the experimental data and the application of MEDRAS in this study—where necrosis-inducing genomic rearrangements are represented as cell death events—is reflected in the calibrated kinetic parameters of the compartment model [12]. The Olaparib treatment was modeled by the adaption of only the constant PARPi survival term $\:{\mu\:}_{PARPi}$, while the Rucaparib adaption was performed by adaption of $\:{\mu\:}_{PARPi}$ and a reduced SSB repair rate $\:{\lambda\:}_{ssb}$. The accurate modeling of Olaparib’s presence through only the PARPi survival term suggests that Olaparib has no radiosensitizing effect on NCI-H69 cells, which is consistent with previous findings [17].

The model generated results that were mostly within experimental errors margins (Fig. 2A, B), with average deviations of 5.3 ± 3.2% for RPT monotherapy, 6.1 ± 4.4% Olaparib combination treatment, and 37 ± 32% for Rucaparib combination treatment. For initial activities of 10 and 21.5 kBq, experimental results of RPT and Rucaparib combination treatment show accelerated survival compared with simulation results. This discrepancy suggests an adapted SSB repair model for Rucaparib treatment, where the SSB repair rate decreases with increasing SSB abundance, which shows a validation accuracy of 12 ± 18%.

To generate accurate results with calibrated kinetic parameters proves the concept of this model to possibly replicate RPT and PARPi combination treatment cell-type specifically. This hypothesis is strengthened by the adaption of the model to the same experiment using the prostate cancer cell lines PC3-PIP and C4-2 with a validation accuracy of 3.3 ± 3.5% and 3.3 ± 2.9%, respectively (Fig. 2C). For both cell lines expressing PSMA, the same parameter values for radiopharmaceutical association and dissociation were used across cell lines. Used parameters of the prostate cancer cell simulations can be found in the *Supplementary Information*. Absorbed dose in cancer cell nuclei is determined by radionuclide concentration, calibrated kinetic parameters, radionuclide decay, therapeutic schedule, geometrical cell properties, and receptor concentration (Fig. 2D). Decreasing absorbed dose per activity is due to the limited free receptors.

### Model functionality insights using variable temporal development over the experimental course

**Fig. 3:**
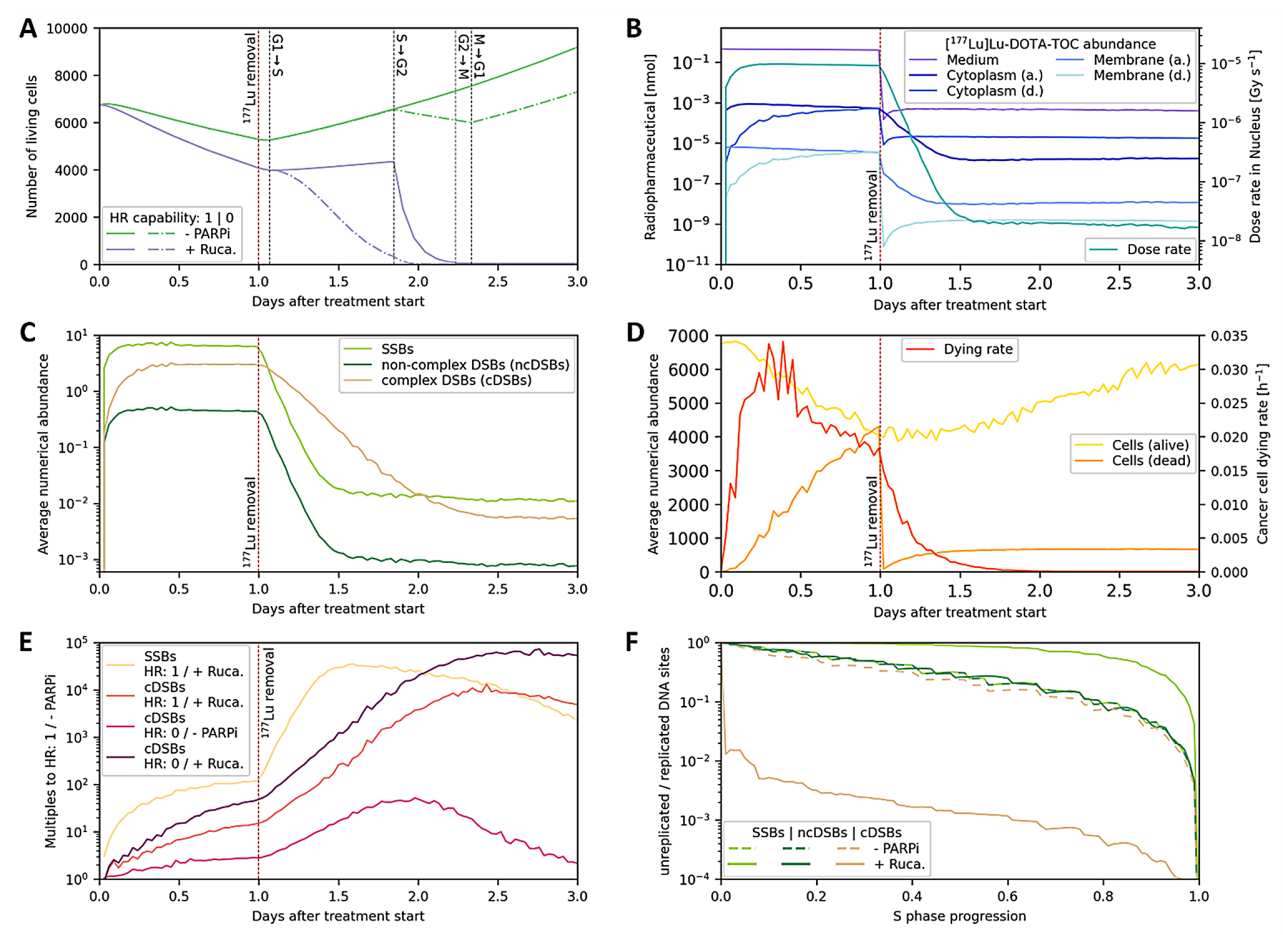
Variable development over experimental time for cells with full HR capability treated with RPT monotherapy ($\:{A}_{0}$: 100 kBq). (**A**) Surviving cells over experimental duration of cells that start into the experiment at the beginning of the G1 phase. (**B**) Development of variables that directly affect the absorbed dose in cellular nuclei from living (a.) and dead (d.) compartments, (**C**) represent DNA lesions, and (**D**) describe cancer cell survival. (**E**) Development of DNA lesion abundance compared with RPT monotherapy with full HR capability. (**F**) DNA lesion ratio during S phases between unreplicated and replicated DNA sites

Differences in cellular radiation response between conditions during specific cell phases can be observed when comparing synchronized cells (Fig. 3A). Additionally, to the generally higher lethality of HRD cells and of additional Rucaparib treatment, the increased lethality in the G2 and M phase under high DSB abundance can be observed, as well as the transition from unreplicated to replicated DNA in case of HRD.

Time courses of different variables (Fig. 3B–D) represent the average of each cell cycle starting point. To obtain the same time points different starting points, data points were interpolated with the adjacent existing time points. Noise arises from discrete nature of cell cycle starting points. Comparison of lesion abundance between different conditions shows a higher effect on the cDSB abundance for Rucaparib combination treatment than for HRD (Fig. 3E). The lesion development during the S phase on sites of different genome number indicates the transformation from SSBs to cDSB during replication (Fig. 3F). Time course analyses were made using the Radau method of the solve_ivp algorithm in SciPy version 1.13.0.

### Experimental protocol is suitable for predicting dose-survival curves

**Fig. 4:**
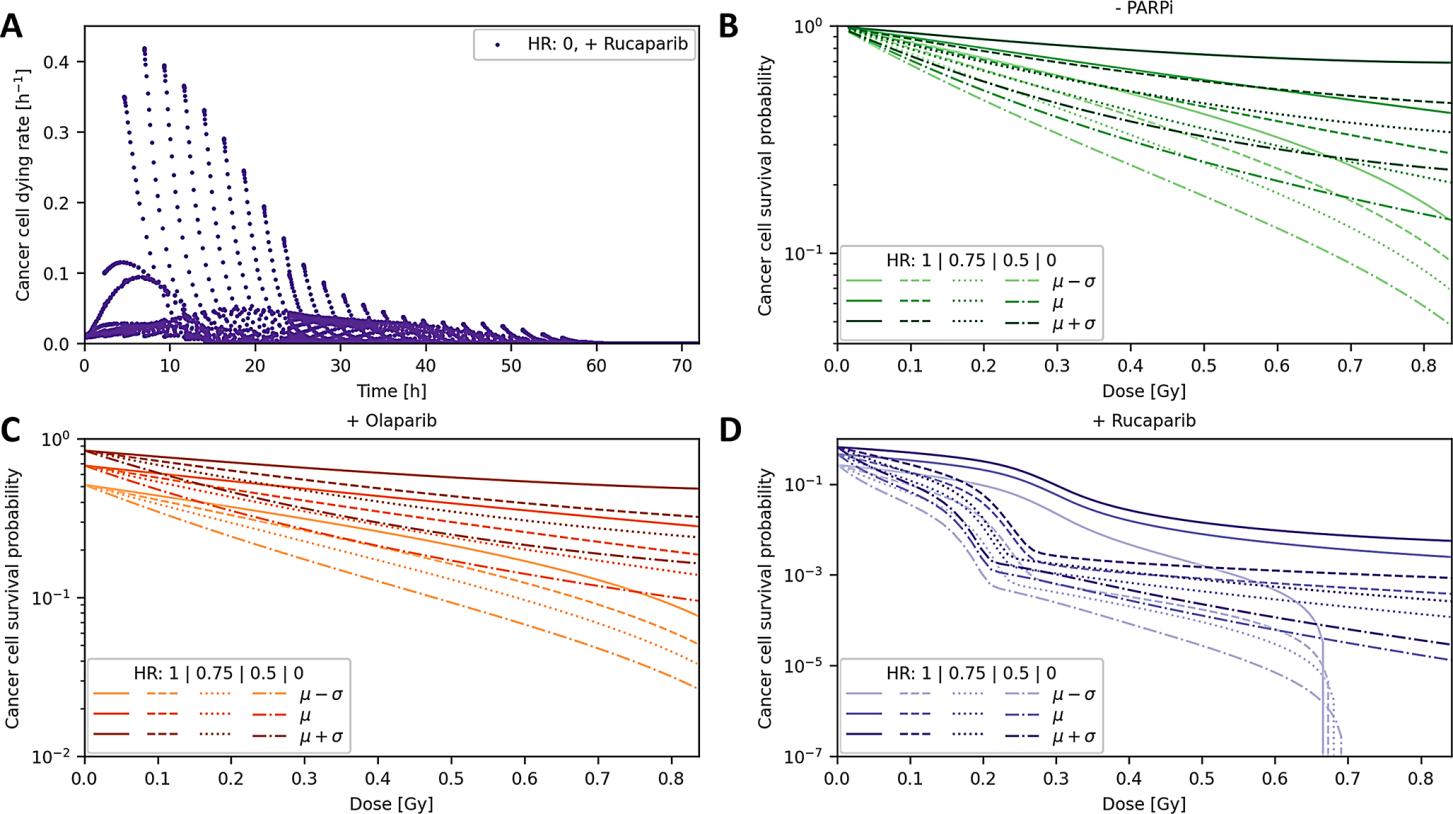
Cell survival probability analysis for different degrees of HR capability based on the absorbed dose in NCI-H69 cell nuclei according to the experimental protocol. (**A**) Collected cancer cell dying rates during the experiment with added initial activity of 100 kBq [^177^Lu]Lu-DOTA-TOC. Each data point represents the average reduction in the cell survival probability across a completed cell phase. Values near to zero at experimental end points show that the experiment is suitable for assessing the cancer cell survival probability. (**B**) Predicted dose-survival curves for [^177^Lu]Lu-DOTA-TOC monotherapy, (**C**) Olaparib combination treatment, and (**D**) Rucaparib combination treatment

The cellular response on therapy must be largely completed when assessing dose-survival over the presented model. Cell survival rates were collected over the experimental duration for the 100 kBq radionuclide condition, suggesting that 48 h post-beta exposure is a suitable time point for assessing survival of NCI-H69 cells (Fig. 4A). Standard deviations were derived from cell culture data, whereby variations for PARPi combination treatments were divided into PARPi monotherapy effects and combination therapy effects.

Dose-survival curves were predicted using therapy simulations (Fig. 4B–D). Given the absence of a radiosensitization effect of Olaparib in NCI-H69 cells, but the presence of a monotherapy effect, we assume in this study that the PARPi monotherapy effect arises through mechanisms other than DSB accumulation. Consequently, it does not exhibit synthetic lethality with HRD, whereas only the radiosensitizing effect of PARPi is associated with synthetic lethality in the context of HRD [17]. The positive curvatures are attributed to the modeled G2/M checkpoint. Under Rucaparib conditions, this effect showed saturation within the investigated dose range due to reduced SSB repair leading to higher DSB abundance. Transition to the quadratic behavior of the LQ model was identifiable for one standard deviation below the mean only. Synthetic lethality between HRD and PARPi is observed in the presence of Rucaparib, as the survival reduction in combined conditions was greater than the product of individual effects. The NCI-H69 cell line was selected for demonstration purposes, as HRD only rarely occurs in small cell lung cancer, such as NCI-H69 (≤ 2% BRCA defects) [18].

### RPT model examined over directed investigations

**Fig. 5:**
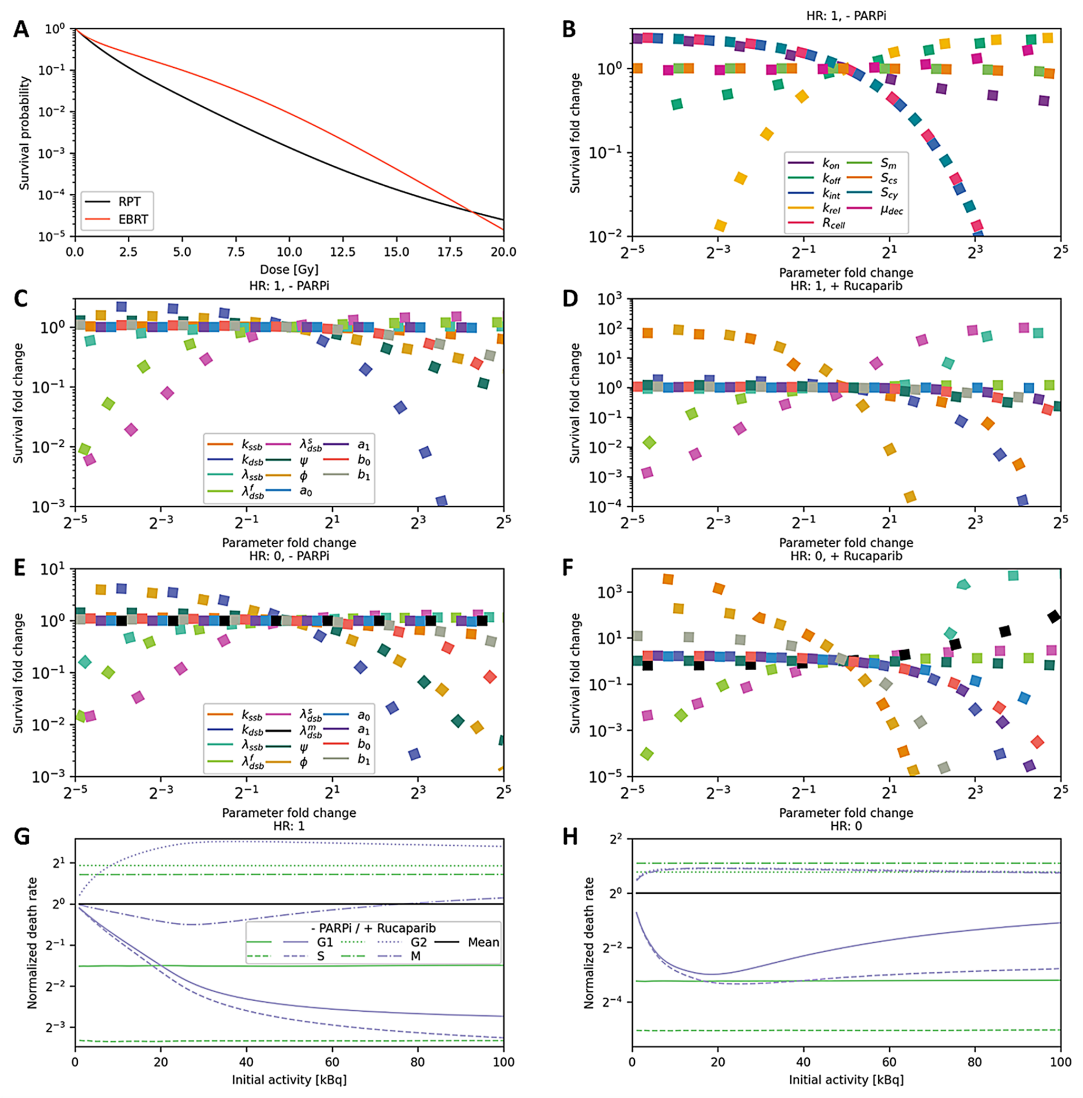
Characteristics of NCI-H69 treatment simulations. (**A**) Comparison of RPT and EBRT both results generated with presented model (**B**) Survival stability analysis for RPT monotherapy ($\:{A}_{0}$: 100 kBq) and full HR capability for varying parameters of the dosimetry model (**C**–**F**) and of the radiobiology model for different treatment and HR status combinations. (**G**) Cancer cell dying rates during cell phases relative to the average survival rate at full HR capability (**H**) and at complete HRD. Legends of subfigures *c*, *e*, and *g* also apply for subfigures *d*, *f*, and *h*, respectively

EBRT is simulated with the presented model over an instantaneous dose delivery at the beginning of the experiment, and RPT is simulated over constant dose deposition over 24 h. RPT shows a higher lethal effect for doses up to 19 Gy, from where EBRT shows higher efficiency (Fig. 5A). The smaller lethality of EBRT for low doses is caused by higher sensitivity of the G2/M checkpoint for higher number of present DSBs and hence, for more immediate characters of radiation. From a certain dose on, effect of misrepair probabilities and genomic loss, which is higher for instantaneous radiation character, overcome the checkpoint effect. That EBRT is more dose efficient than RPT has previously been stated [19].

However, the higher efficiency of RPT for lower doses has not been reported in comparative studies [19]. The G2/M checkpoint model used here assumes a threshold of DSB abundance to assess mitotic catastrophe, rather than explicitly representing cell cycle arrest. This simplification likely explains deviations from prior comparisons. Our findings suggest that the mitotic catastrophe parameter, adopted from the MEDRAS framework, may lose physiological accuracy in the context of an RPT model [12]. A more realistic approach might involve modeling actual cell cycle arrest to account for the varying dose rates characteristic for RPT.

The synchronous survival stability analysis graphs of the parameters $\:{k}_{int},\:{R}_{cell},$ and $\:{S}_{cy}$ concerning the dosimetry model, suggests that SSTR2 receptors are nearly fully occupied under initial activity of 100 kBq in considered system (Fig. 5B). This finding is supported by the experimental data, showing that the number of surviving NCI-H69 cells shows saturation when the initial activity approaches 100 kBq. Further, the nearly static behavior of the $\:{S}_{m}$ and$\:\:{S}_{cs}$ graphs imply insignificant membrane and medium effects. The apoptosis mechanism, which especially is significant RPT monotherapy (Fig. 5C-F), is expected to have a smaller impact on actual NCI-H69 cells due to its p53 gene defect [20]. The cell death mechanism distribution depicts a cell type average.

Radiosensitivity is compared among cell phases based on survival rates (Fig. 5G, H). The RPT model shows the lowest cancer cell death rates during the G1 and S phases and the highest during the G2 and M phases. These results align with reported outcomes [21]. The presence of Rucaparib alters the relative radiosensitivity of cell phases over initial activity, as the conversion effect becomes more significant at higher doses.

### Preliminary extension to whole body considerations

**Fig. 6:**
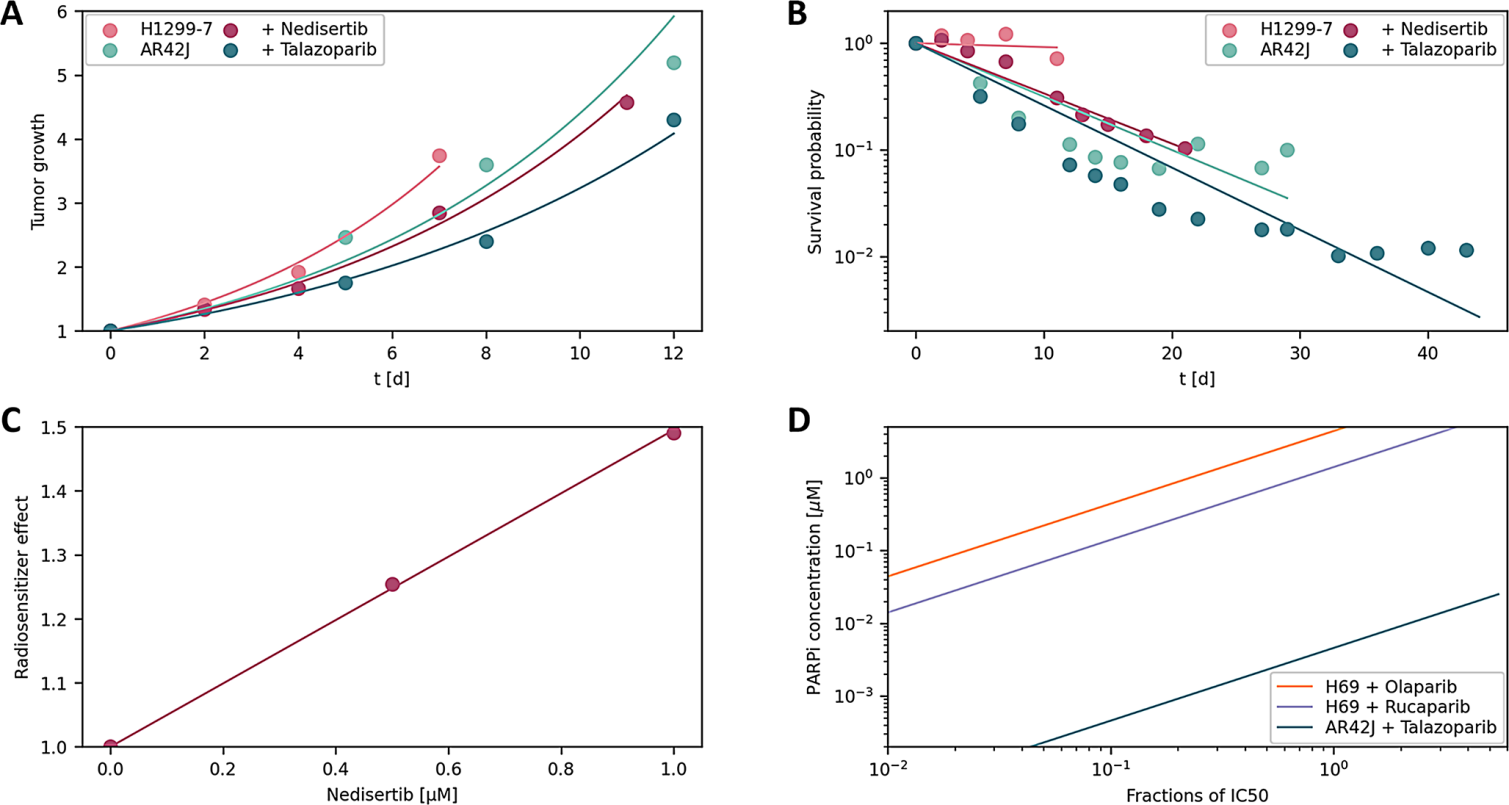
Integration of in vivo data into the presented model, comparison with the model calibrated to in vitro data (**A**) Tumor growth model assuming exponential cell growth. $\:{\mu\:}_{gr}$(H1299-7, AR42J): 0.182 d^-1^, 0.148 d^-1^. $\:{\mu\:}_{PARPi}$(Nedisertib Talozoparib): 0.959 d^-1^, 0.969 d^-1^ (**B**) Cell survival probabilities of tumor cells predicted by the model. $\:{\mu\:}_{dea}$(H1299-7: [^177^Lu]Lu monotherapy, Nedisertib combination treatment; AR42: [^177^Lu]Lu monotherapy, Talazoparib combination treatment): 8.30∙10^− 3^ d^-1^, 0.108 d^-1^, 0.115 d^-1^, 0.134 d^-1^ (**C**) Radiosensitizer effect showing linear behavior across the applied concentration range in an in vitro experiment. (**D**) Comparison of PARPi radiosensitizer effects between data-integrated models from in vivo experiments (AR42J) and in vitro experiments (H69)

In vivo data from [^177^Lu]Lu-DOTA-TATE treated tumor-bearing mice were integrated into the presented computational model [3, 22]. Those xenograft experiments utilized Talazoparib as PARPi radiosensitizing agent. Exponential growth is assumed and radiosensitizer monotherapy effects are determined by inserting Eq. 11 into Eq. 12 (Fig. 6A). Solving this differential equation results in $\:{N}_{alive}\left(t\right)={N}_{alive}\left(0\right){e}^{{\mu\:}_{gr}-{\mu\:}_{PARPi}+1}$. Survival values are obtained numerically by using the same equations in inverse, resulting in $\:{d}_{t}S=-\left({\mu\:}_{gr}-\frac{{d}_{t}{N}_{cell}}{{N}_{cell}}\right)S$ (Fig. 6B). This model relates cancer cell death rate to cell survival, represented by $\:S={e}^{-{\mu\:}_{dea}t}$ as solution of Eq. 11.

To find a correlation between radiosensitizer concentration and effect, in vitro data of treated H1299-7 cells were processed similarly to the in vivo data. The cell death rate ratios, relative to the absence of the radiosensitizer Nedisertib, represent the radiosensitizer effect and are the mean of the different radionuclide concentrations. These data points form a near-linear relationship (Fig. 6C), suggesting that radiosensitizer effects increase linearly with concentration within the therapeutic window. A concentration ratio between tumor microenvironment and the whole body of 7.2∙10^− 3^ is derived and assumed universally applicable.

The graphs in Fig. 6D correlate the concentrations of different PARPi agents with their respective IC50 values where the suggested linear relationship between radiosensitizer effect and concentration within the therapeutic window is assumed. As noted, Olaparib exhibits a low effect since only the monotherapy effect is evident when treating H69 cells. The observed order of PARPi effects is consistent across both in vitro and in vivo data and aligns with published results [23]. Olaparib and Rucaparib have been reported to exhibit similarly strong effects, while Talazoparib shows a significantly stronger effect. This also suggests that the range of inhibitor strengths obtained in this study aligns with previously reported data.

### Strong olaparib effect predicted on cancers harboring mutations associated with HRD

**Fig. 7:**
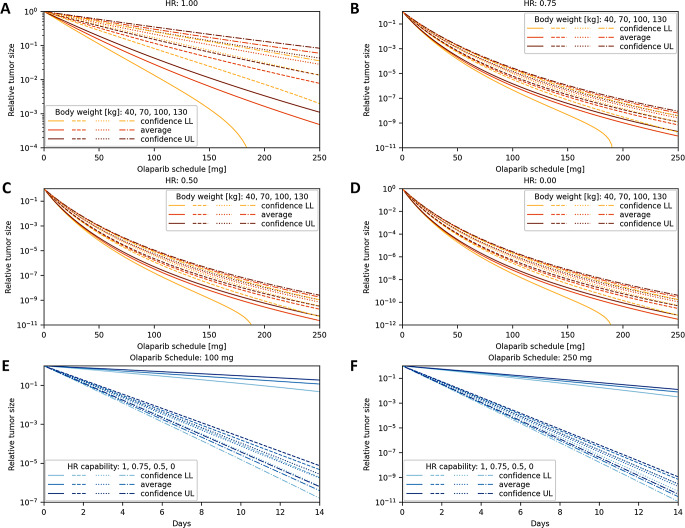
*In silico* testing of the LuPARP protocol predicts Olaparib effects based on in vivo data integration. Tumor sizes are relative to [^177^Lu]Lu-PSMA-617 monotherapy. (**A**) Olaparib effects after 14 days of lutetium treatment for full HR capability, (**B**) HR capability of 75%, (**C**) HR capability of 50%, (**D**) and complete HRD. (**E**) Progression of Olaparib effects after therapy start for a patient with a body weight of 70 kg under administration of 100 mg Olaparib (**F**) and 250 mg Olaparib. A normal distribution for each Olaparib amount and time after therapy start, respectively, represents the stochastic behavior of the tumor size with mean $\:\mu\:$ and confidence interval of level 95% with lower limit (LL) and upper limit (UL)

Building on the LuPARP trial, where metastatic castration-resistant prostate cancer patients are treated with a combination of [^177^Lu]Lu-PSMA-617 and Olaparib, this study predicts Olaparib effects based on patient body weight and degree of HRD [24]. Utilizing advancements from preliminary in vivo studies, Olaparib effects are forecasted. Given that NCI-H69 cells respond differently to Olaparib than most cells, and considering that the radiosensitizing effects of Olaparib and Rucaparib are similar across most cell types, the Rucaparib model is employed to predict Olaparib effects [17, 23] Confidence intervals were derived from cell culture data.

The results indicate that Olaparib’s efficacy significantly depends on patient body weight (Fig. 7A). To predict Olaparib effects in HRD cancer cells, the Olaparib effect after three days is mapped to the average initial activity of 25.9 kBq, adjusted for body weight using previously used data-integrated model. Olaparib effects in HRD cells are derived. The findings show that Olaparib’s effect increases drastically in HRD cancer cells, with the degree of HRD playing a secondary role (Fig. 7B–D). Olaparib’s effect over time is calculated based on its radiosensitizing effect, represented as the inverted cancer cell death rate in this context. A significant effect of Olaparib scheduling is observed, as well as mutations in HRD-related genes. The obtained results are preliminary and predictive in nature and do not quantitatively align with previously published in vivo data [25].

## Discussion

This study presents the first computational model for simulating the combined treatments of RPT and PARPi. The model comprises a dosimetry component and a radiobiological response component, where DNA lesions are represented and their impact on cell survival is assessed for varying degrees of HR capability and with PARPi combination treatment. Integration of in vivo data allows for comparison of radiosensitizer effects between in vitro and xenograft experiments, facilitating preliminary predictions of clinical protocol outcomes. Future efforts incorporating physiological drug distribution are ongoing and will further improve the accuracy of in *vivo* predictions considering blood flow and tumor microenvironment heterogeneity, enabling closer alignment with previously published preclinical in vivo data [25–27].

The cellular response to RPT extends beyond the experimental duration selected for this study [28]. As the presented model evaluates cell fate concurrently with DSB repair, the cellular response is nearly complete by the end of the observation period. This leads to a bias in the kinetic parameter values; however, the results remain meaningful. The cellular response in actual cells occurs over a longer timescale. Future studies could further investigate the temporal discrepancy between the occurrence of necrosis-inducing genomic rearrangements and cell death. Other long-term therapeutic effects such as senescence encompass cellular responses that extend beyond modeled radiation-induced DNA damage consequences. These effects are implicitly accounted for in the MEDRAS radiobiology model, not as a distinct state but through parameter calibration based on experimental data that inherently includes these effects [12]. While explicitly modeling senescence could further refine the accuracy of the model, the chosen approach prioritizes simplicity while ensuring sufficient precision for the objectives of this study. Additionally, the 24-hour treatment duration does not reflect clinical conditions but was selected in this proof-of-concept study to emphasize differences between the analyzed conditions. This method is unbiased in obtaining results; however, future studies could incorporate clinical treatment durations to further enhance its clinical accuracy.

Like all law-driven models, the one presented here is inherently overparameterized, which carries the risk of overfitting. Although the model requires a substantial number of parameters (up to 26 per condition), the parameter value estimation process was carefully designed to minimize overfitting while ensuring the most reasonable values. As a result of this structured approach, only four parameters needed to be fitted per step. The relatively large overall number of parameters is necessary because the model serves as a framework for describing RPT in combination with PARPi treatment while also accounting for HRD. This study’s efforts suggest that the presented model includes a minimum number of parameters required to simultaneously represent these conditions. Although most kinetic parameters fitted in this study have not been validated against empirical metrics across different cell lines, they remain useful for simulating results that serve as a proof-of-concept for the presented model. Future clinical studies could use existing quantitative data to further refine the physiological relevance of the kinetic parameter values. Parameters with a high margin of error limit the full potential of the presented model. While the parameters used in this study generated accurate results for proof-of-concept, future clinical studies focused on optimizing these parameter values would further enhance the model’s physiological applicability. Despite its considerable complexity, the model is designed to maintain a necessary balance between simplicity and the ability to accurately capture key aspects of therapy response. Done efforts to identify the simplest mechanistic approach capable of capturing the required framework led to the development of the presented model. For practical clinical applications, future efforts to simplify the model could utilize insights from the parameter stability analysis to exclude negligible effects under the target conditions. As the presented model simulates therapeutic outcomes on a preliminary basis, it is poised for clinical validation in future studies focused on specific clinical applications. Additionally, the model has the potential to generate hypotheses that can be experimentally tested. Key areas already addressed by the model include estimating RPT/PARPi combination treatment effects, assessing HRD/PARPi synthetic lethality, predicting tissue toxicity, optimizing clinical protocols, and minimizing patient radiation exposure. Further refinements will enhance the model’s precision in these applications.

The model integrates established RPT-specific dosimetry with dose-dependent cancer cell survival [12, 26]. Originally designed for EBRT, the radiobiological cell survival model requires immediate or discrete dose delivery, which is incorporated as stepwise dose impacts in short intervals. Radiobiological parameters are adopted from X-ray studies due to the similar LET between X-rays and β particles [12, 19]. An adaption to alpha therapy would require a different calibration of the radiobiological parameters. HRD is frequently observed in prostate cancer; therefore, PARP inhibitor–mediated radiosensitization, particularly with Rucaparib, is expected to be highly effective in this cancer type [29]. The spherical cell geometry model was chosen to effectively represent cellular geometry within the complex and highly parameterized framework, particularly as NCI-H69 cells exhibit a nearly spherical shape (*Supplementary Information*). This model served its purpose for the proof-of-concept presented in this study. Future studies focused on optimizing microdosimetry may benefit from using more detailed cell shapes.

The results related to PARPi concentrations are initial assessments, as they are based on a simplified drug distribution model. The PARPi effect in the model is based on reduced SSB repair capability, leading to accelerated conversion of SSBs to DSBs. Although this model is a simplified representation of PARPi mechanisms, it effectively recreates experimental outcomes in cell culture [4]. Recent debates on synthetic lethality between PARPi and HRD suggest that more severe effects of PARP traps occur in HRD cells due to stalled replication forks that cannot restart in the absence of functional HR repair [23]. Also, the incorporation of PARPi resistance mechanisms of tumor cells to evade PARPi effects, PARPi concentrations effects, or further, by considering molecular mechanisms of specific PARPi types could contribute to accurate treatment depictions [23]. The latter could guide the selection of the PARPi type based on the specific cancer subtype and the radiopharmaceutical used and possibly giving insights about the unique response of NCI-H69 cells on Olaparib radiosensitization [17]. The applicability of this proof-of-concept study is limited by the small number of cell lines and PARP inhibitor types. However, the selected cell lines remain relevant, and the included PARP inhibitors are widely used. Expanding the model to incorporate additional cell lines and PARP inhibitors could enhance its applicability and enable systematic validation in future studies. The model’s design also enables an initial exploration of quantitative relationships in emerging therapeutic strategies, such as DNAPKcs and CDK4/6 inhibitors, by requiring modifications to only a limited set of parameter values.

## Conclusion

The presented RPT model successfully reproduces experimental in vitro data, including scenarios involving PARPi. By demonstrating accuracy in in vitro systems and providing preliminary whole-body and dose-dependent PARPi predictions, the model sets the stage for data-driven enhancements to accurately represent treatments in patients, incorporating realistic PARPi concentration effects. Currently, the study addresses radiotherapy within a constant microenvironment, with the integration of realistic tissue effects planned for subsequent phases. This study marks a relevant step toward developing digital twins for optimizing cancer treatment protocols involving the combination of RPT and PARPi therapy.

## Electronic supplementary material

Below is the link to the electronic supplementary material.

Supplementary Material 1

## Data Availability

The Pythons script used to perform the simulations and generate the results can be downloaded at https://github.com/Marc-Ryhiner/RPT-PARPi-Model.
