# Supplementary material for: A mathematical model for the investigation of combined treatment of radiopharmaceutical therapy and PARP inhibitors
Source: Eur J Nucl Med Mol Imaging. 2025 Feb 20;52(9):3252–65. doi: 10.1007/s00259-025-07144-y (PMC12222425; doi:10.1007/s00259-025-07144-y)
Supplement: Supplementary file 1 — Supplementary Material 1 [file 259_2025_7144_MOESM1_ESM.pdf]

Title: A Mathematical Model for the Investigation of Combined Treatment of Radiopharmaceutical Therapy and PARP inhibitors

Journal: European Journal of Nuclear Medicine and Medical Imaging

Authors: Marc Ryhiner[1], Yangmeihui Song[2], Jimin Hong[1], Carlos Vinícius Gomes Ferreira[1], Axel Rominger[1], Susanne Kossatz[2], Gerhard Glatting[3], Wolfgang Weber[2], Kuangyu Shi[1]

Affiliation:

1. Department of Nuclear Medicine, Inselspital, University of Bern, Bern, Switzerland
2. Department of Nuclear Medicine, Klinikum rechts der Isar, Technical University of Munich, Munich, Germany
3. Medical Radiation Physics, Department of Nuclear Medicine, Ulm University, Ulm, Germany

Correspondance: Marc Ryhiner: marc.ryhiner@unibe.ch

Variable settings and parameter values

Table S1 Non-zero initial variable values and functions for RPT simulation.

Symbol	Variable	Initial value / function
$c_m(0\ h)$	[^{177}Lu]Lu-DOTA-TOC concentration in medium	$4.55\cdot10^{-8}\cdot A_0$ nmol Bq^{-1} mL^{-1}
$A(0\ h)$	Activity from [^{177}Lu]lutetium within the *in vitro* system	A_0
$S(0\ h)$	Cell survival probability	1
$N_{alive}(0\ h)$	Number of living cells within the *in vitro* system	$6.742\cdot10^3$

Table S2 Abruptly changing variable values and functions due to radionuclide removal during RPT simulation.

Symbol	Variable	Adapted value / -function
$c_m(24\ h)$	[^{177}Lu]Lu-DOTA-TOC concentration in medium	0 nmol mL^{-1}
$c_{dcs}(24\ h)$	[^{177}Lu]Lu-DOTA-TOC concentration on surfaces of dead cells	0 nmol mL^{-1}
$c_{dcy}(24\ h)$	[^{177}Lu]Lu-DOTA-TOC concentration in cytoplsm of dead cells	0 nmol mL^{-1}
$A(24\ h)$	Radionuclide activity	$A(t)\dfrac{c_{acs}(t)+c_{cy}(t)}{c_m(0\ h)}$
$N_{dead}(24\ h)$	Number of dead cells within *in vitro* system	0

Table S3 Parameter values used for RPT microdosimetry model with constant microenvironent.

Symbol	Parameter	Value
k_{on}	Association rate [^{177}Lu]Lu-DOTA-TOC ↔ SSTR2	40 mL nmol^{-1} s^{-1}
k_{off}	Dissociation rate [^{177}Lu]Lu-DOTA-TOC ↔ SSTR2	0.2 s^{-1}
k_{int}	Integration rate [^{177}Lu]Lu-DOTA-TOC-SSTR2 ↔ NCI-H69	0.03 s^{-1}
k_{rel}	Release rate [^{177}Lu]Lu-DOTA-TOC-SSTR2 ↔ NCI-H69	$2\cdot10^{-4}$ s^{-1}
R_{cell}	STTR2 concentration	$2\cdot10^{-11}$ nmol mL^{-1} cell^{-1}
S_m	Dosimetry S value (nucleus ← medium/crossfire)	$2.43\cdot10^{-9}$ Gy Bq^{-1} s^{-1}
S_{cs}	Dosimetry *S* value (nucleus ← living cell surface)	$3.27\cdot10^{-4}$ Gy Bq^{-1} s^{-1}
S_{cy}	Dosimetry *S* value (nucleus ← living cytoplasm)	$3.27\cdot10^{-4}$ Gy Bq^{-1} s^{-1}
μ_{dec}	[^{177}Lu]lutetium decay constant	$1.207\cdot10^{-6}$ s^{-1} [1]

Table S4 Parameter values used for cell cycle implications.

Symbol	Parameter	Value
μ_{inter}	NCI-H69 S phase progression rate	$1.48800\cdot10^{-5}$ s^{-1} [2]
μ_{gr}	NCI-H69 growth rate	$3.4382\cdot10^{-6}$ s^{-1} [2]

Table S5 Parameter values and functions used for RPT radiobiology and PARPi response model. p_{dsb}^{s} and p_{dsb}^{m} are the DSB proportions of slow and MMEJ kinetics among overall DSBs, respectively.

Symbol	Parameter	Value / Function
N_{gen}	Number of present genomes	
	• G1	1
	• S	$1 + s_{prog}$
	• G2/M	2
k_{ssb}	Radiation-induced SSBs	$1 \cdot 10^{3}$ SSB Gy^{-1} $genome^{-1}$ [3]
k_{dsb}	Radiation-induced DSBs	35 DSB Gy^{-1} $genome^{-1}$ [4]
λ_{ssb}	SSB repair rate	
	• - PARPi / + Olaparib	0.00201 s^{-1} [5]
	• + Rucaparib	$2 \cdot 10^{-6}$ s^{-1}
	(alternative)	$5 \cdot 10^{-5}\ e^{-\lambda_{ssb}^{red} N_{ssb}(t)}$ s^{-1}
λ_{ssb}^{red}	SSB repair reduction rate	0.013 SSB^{-1}
λ_{dsb}^{f}	DSB fast repair kinetics rate	$5.78 \cdot 10^{-4}$ s^{-1} [4]
λ_{dsb}^{s}	DSB slow repair kinetics rate	$7.23 \cdot 10^{-5}$ s^{-1} [4]
λ_{dsb}^{m}	DSB MMEJ repair kinetics rate	$2.35 \cdot 10^{-6}$ s^{-1} [4]
p_{c}	Complexity probability beta radiation-induced DSB	0.43 [4]
μ_{PARPi}	PARPi survival rate	
	• - PARPi	1 s^{-1}
	• + Olaparib	0.9999985 s^{-1}
	• + Rucaparib	0.9999975 s^{-1}
	(alternative)	0.9999970 s^{-1}
ψ	Apoptosis rate	0.0162 DSB^{-1} [4]
φ	Mitotic catastrophe rate	$6.27 \cdot 10^{-6}$ DSB^{-1} s^{-1} [4]
a	Quadratic survival probability term due to genomic loss	
• a_{0}	• Unreplicated DNA	$(2.04543 \cdot 10^{-8} \cdot p_{dsb}^{s}(t)$ - $2.34390 \cdot 10^{-8})$ DSB^{-2} [4]
• a_{1}	• Replicated DNA	$(3.01620 \cdot 10^{-6} \cdot p_{dsb}^{s}(t)$ + $3.01083 \cdot 10^{-6} \cdot p_{dsb}^{m}(t)$ - $3.02302 \cdot 10^{-6})$ $4.02205 \cdot 10^{-3}$ DSB^{-2} [4]
b	Linear survival probability term due to genomic loss	
• b_{0}	• Unreplicated DNA	$(1.94923 \cdot 10^{-6} \cdot p_{dsb}^{s}(t)$ - $2.21282 \cdot 10^{-6})$ DSB^{-1} [4]
• b_{1}	• Replicated DNA	$(4.43926 \cdot 10^{-4} \cdot p_{dsb}^{s}(t)$ + $4.36892 \cdot 10^{-4} \cdot p_{dsb}^{m}(t)$ - $4.42982 \cdot 10^{-4})$ 0.0122819 DSB^{-1} [4]

Parameter a_0, b_0, a_1, and b_1, calibration using MEDRAS [4]

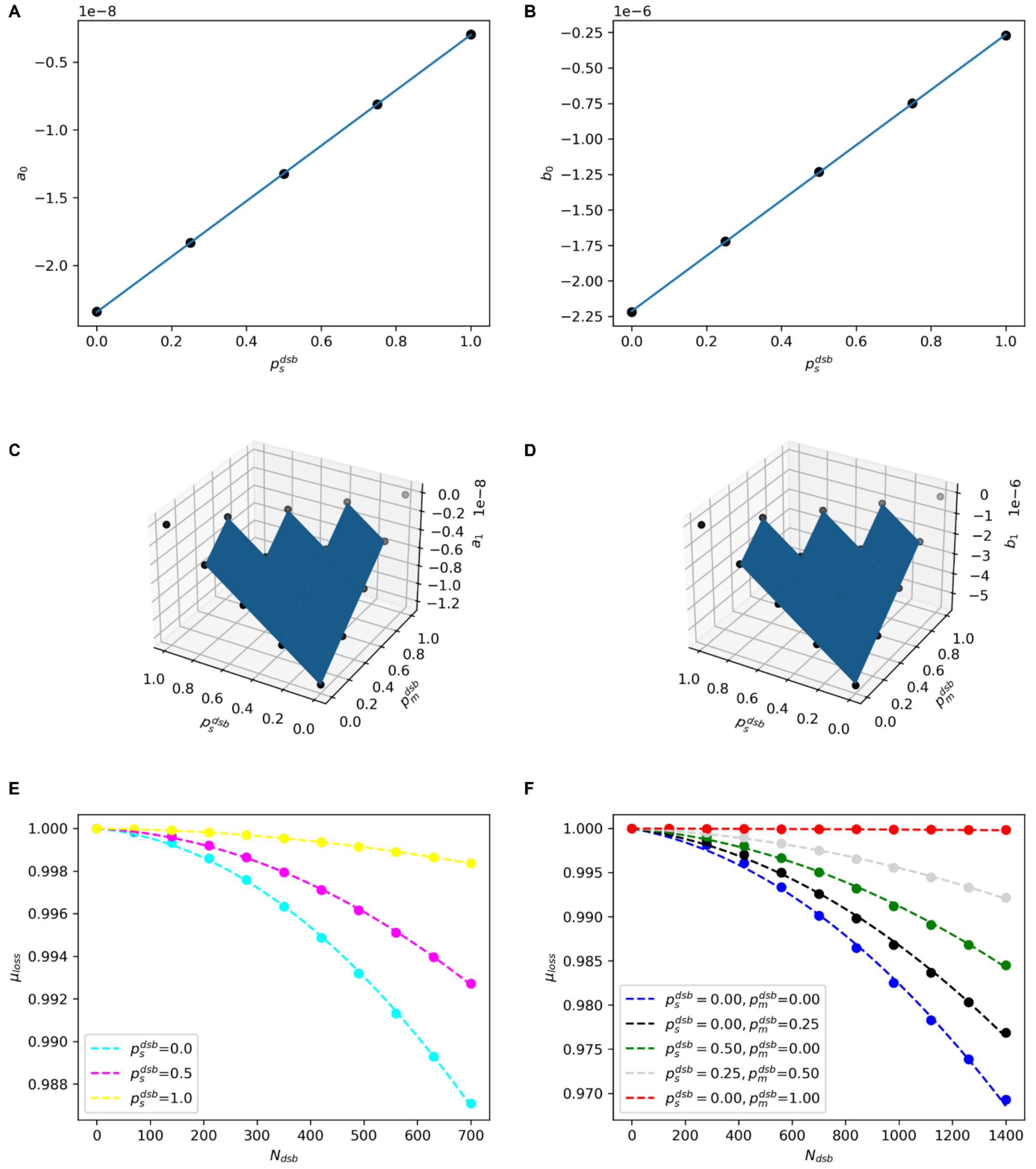

Figure S1 Calibration of the linear and quadratic parameters for survival due to genomic loss [6]. (*A*) Calibration of the quadratic term in presence of one genome a_0: m: 2.04543090·10^{-8} DSB^{-2}; c: -2.34389810·10^{-8} DSB^{-2}, (*B*) of the linear term in presence of one genome b_0: m: 1.94922930·10^{-6} DSB^{-1}; c: -2.21282442·10^{-6} DSB^{-1}, (*C*) of the quadratic term in presence of two genomes a_1: k: -3.01620264·10^{-6} DSB^{-2}; l: -3.01083147·10^{-6} DSB^{-2}; m: 248.629736 DSB^{-2}; n: 3.02301534·10^{-6} DSB^{-2}, (*D*) and of the linear term in presence of two genomes Parameters: b_1: -4.43925698·10^{-4} DSB^{-1}; l: -4.36891635·10^{-4} DSB^{-1}; m: 81.4203768 DSB^{-1}; n: 4.42982208·10^{-4} DSB^{-1}. (*E*) Survival rates due to genomic loss for example DSB repair kinetic proportions for unreplicated genomes and (*F*) for replicated genomes. Dots represent MEDRAS simulated results [4], and dashed lines represent survival rate functions according to characteristic a and b.

Survival due to genomic loss is modeled to follow a quadratic relationship as a function of doble-strand break (DSB) abundance. Given that repair pathways vary in their kinetics and fidelity, the parameters for the survival

rate in the context of genomic loss depend on the proportions of involved DSB repair pathways. The calibrations differ for unreplicated and replicated genomes due to the potential of homologous recombination (HR) failure in conditions of HR deficiency (HRD) and the different implications of genomic loss when an additional genome is present. Since repair kinetics are synchronized with repair pathways within boundaries of equal genomic abundance, the parameters for the survival rate due to genomic loss are calibrated according to the proportions of repair kinetics.

Using MEDRAS [4], aberration survival one second after exposure is simulated for the G1 phase over a dose range from zero to twenty gray, considering different probabilities of DSB complexity, which correspond to proportions of slow repair kinetics. The simulation results are listed in Table S1. Simulations for the G2 phase are conducted in the same manner but also account for varying probabilities of HR failure. Combined probabilities of DSB complexity and HR failure correspond to proportions of MMEJ kinetics, with the results provided in Table. S2.

The simulated aberration survivals are used to calibrate a and b values of the function $\mu_{loss} = [aN_{dsb} + b]N_{dsb} + 1$. These a and b values form perfect line when plotted against slow repair kinetic proportions during the G1 phase, and a perfect plane when plotted against both slow and MMEJ repair kinetic proportions during G2 the phase. Consequently, the quadratic parameters for the presence of one genome represented by the linear equation $mp_s + c$, while for the presence of two genomes, they are represented by the plane equation $-\frac{kp_s+lp_m+n}{m}$.

Finally, the parameters these linear and planar equations are derived by calibrating them against the a and b values. This two-step calibration process is performed using the curve_fit algorithm from scipy.optimize (version 1.13.0). The calibration assumes that 1 Gy induces 35 DSBs per genome.

Calculation of receptor concentration and radionuclide molarity

The receptor concentration contributed by a single cell is calculated based on the membrane mass and the receptor molarity per unit of membrane mass (Eq. 1). To determine the membrane mass, the membrane is assumed to be spherical, with half of the membrane mass attributed to membrane proteins [7].

$$R_{cell} = \frac{8\pi r^2 \rho M_{prot}^{mem} c}{N_A V} \qquad (1)$$

where r is the cell radius, ρ is the average number of membrane proteins per unit membrane area ($3 \cdot 10^4$ μm^{-2} [8]), M_{prot}^{mem} is the average molar mass of membrane proteins (48 g $mmol^{-1}$ [9]), N_A is the Avogadro constant, c is the molarity of the specific receptor within the membrane of interest (for SSTR2 in NCI-H69 cell plasma membranes: 0.721 nmol g^{-1} [10]), and V is the system volume of 0.1 mL.

The initial molarity of the radionuclide in the medium is calculated using the initial activity and specific activity, expressed by the equation $c_m(0\ h) = \frac{A_0}{A_m}$, where A_m is the molar activity, which is 220 MBq $nmol^{-1}$ in this study.

Table S6 Simulated aberration survival in the G1 phase using MEDRAS [4], measured 1 second after exposure.

p_s	0 Gy	2 Gy	4 Gy	6 Gy	8 Gy	10 Gy	12 Gy	14 Gy	16 Gy	18 Gy	20 Gy
0.00	1.000000	0.999811	0.999339	0.998595	0.997591	0.996344	0.994871	0.993192	0.991325	0.989289	0.987102
0.25	1.000000	0.999852	0.999484	0.998902	0.998117	0.997142	0.995990	0.994676	0.993215	0.991621	0.989908
0.50	1.000000	0.999893	0.999628	0.999209	0.998644	0.997941	0.997111	0.996164	0.995110	0.993959	0.992722
0.75	1.000000	0.999935	0.999773	0.999517	0.999171	0.998741	0.998233	0.997653	0.997008	0.996303	0.995545
1.00	1.000000	0.999976	0.999917	0.999824	0.999698	0.999542	0.999356	0.999145	0.998910	0.998653	0.998376

Table S7 Simulated aberration survival in the G2 phase using MEDRAS [4], measured 1 second after exposure.

p_s / p_m	0 Gy	2 Gy	4 Gy	6 Gy	8 Gy	10 Gy	12 Gy	14 Gy	16 Gy	18 Gy	20 Gy
0.00 / 0.00	1.000000	0.999495	0.998159	0.996078	0.993364	0.990129	0.986477	0.982499	0.97827	0.973851	0.969292
0.00 / 0.25	1.000000	0.999621	0.998617	0.997055	0.995015	0.992583	0.989835	0.986838	0.983649	0.980314	0.976869
0.00 / 0.50	1.000000	0.999742	0.999066	0.998017	0.996648	0.995016	0.993170	0.991156	0.989011	0.986765	0.984443
0.00 / 0.75	1.000000	0.999865	0.999520	0.998988	0.998295	0.997468	0.996534	0.995514	0.994427	0.993287	0.992108
0.00 / 1.00	1.000000	0.999988	0.999975	0.999960	0.999944	0.999927	0.999909	0.999891	0.999872	0.999853	0.999834
0.25 / 0.00	1.000000	0.999621	0.998619	0.997057	0.995019	0.992587	0.989840	0.986845	0.983657	0.980323	0.976879
0.25 / 0.25	1.000000	0.999745	0.999073	0.998027	0.996662	0.995034	0.993192	0.991183	0.989043	0.986802	0.984484
0.25 / 0.50	1.000000	0.999868	0.999527	0.998998	0.998309	0.997486	0.996556	0.995541	0.994458	0.993324	0.992150
0.25 / 0.75	1.000000	0.999991	0.999981	0.999970	0.999958	0.999945	0.999932	0.999918	0.999904	0.999890	0.999875
0.50 / 0.00	1.000000	0.999748	0.999079	0.998037	0.996676	0.995052	0.993215	0.991210	0.989074	0.986838	0.984525
0.50 / 0.25	1.000000	0.999871	0.999533	0.999008	0.998323	0.997505	0.996579	0.995568	0.994490	0.993360	0.992191
0.50 / 0.50	1.000000	0.999994	0.999987	0.99998	0.999972	0.999964	0.999955	0.999946	0.999936	0.999927	0.999917
0.75 / 0.00	1.000000	0.999874	0.999539	0.999018	0.998337	0.997523	0.996602	0.995595	0.994522	0.993397	0.992232
0.75 / 0.25	1.000000	0.999997	0.999994	0.99999	0.999986	0.999982	0.999977	0.999973	0.999968	0.999963	0.999958
1.00 / 0.00	1.000000	1.000000	1.000000	1.000000	1.000000	1.000000	1.000000	1.000000	1.000000	1.000000	1.000000

Dosimetry S value estimations

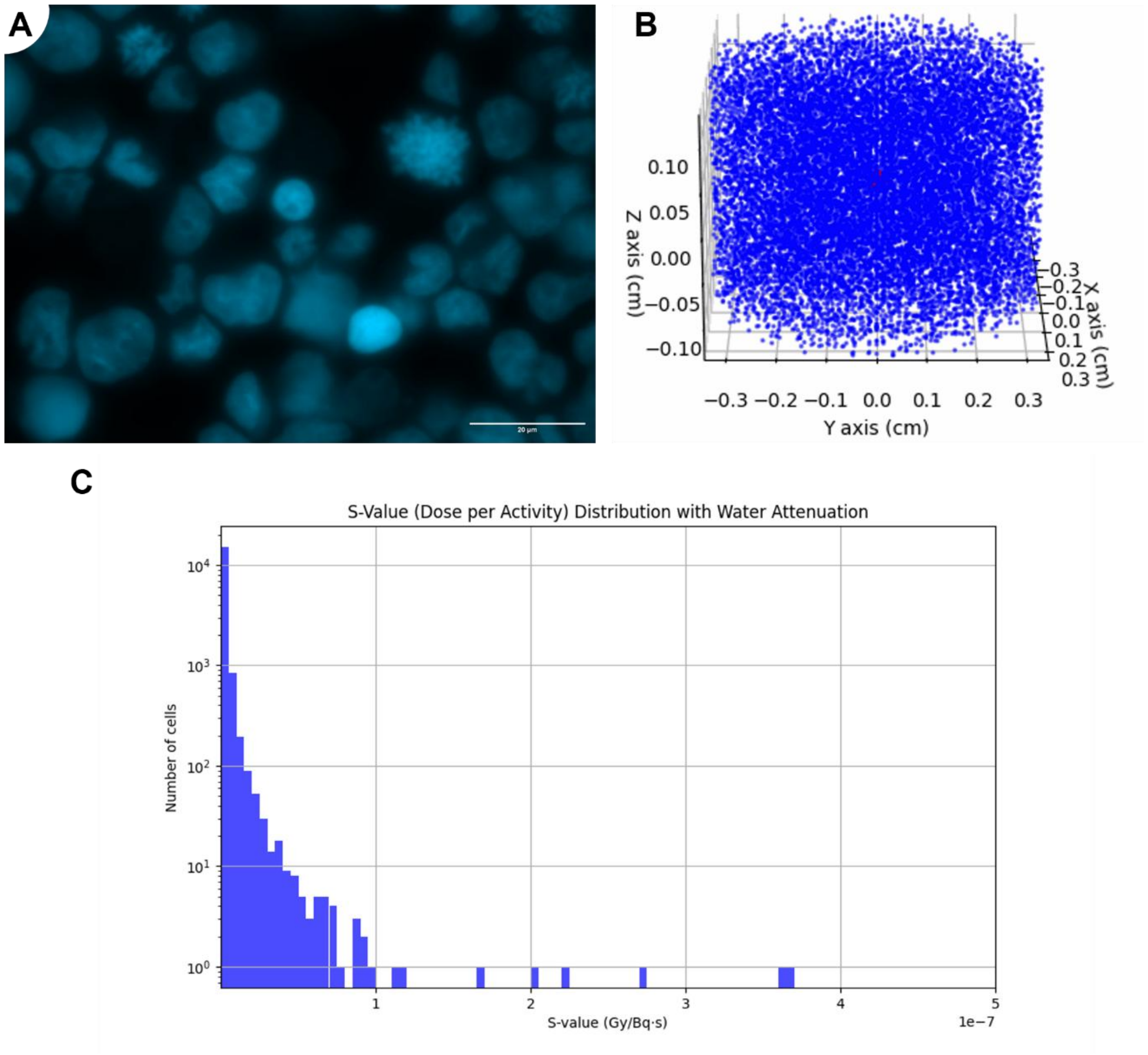

Figure S2 Reasoning for S values (S_m, S_{cs}, S_{cy}) estimations (*A*) Microscopic image of DAPI-stained DNA of NCI-H69 cells. Little spacing between DNA of adjacent cells on the horizontal/vertical plane suggests thin cytoplasm shells. (*B*) Simulated well containing 16'407 evenly spaced representations of NCI-H69 cells (cylinder radius: 0.343 cm, cylinder height: 0.271 cm, cell radius: 6 μm, sourve energy: 140 keV, source activity: 1 MBq, attenuation coefficient: 0.153 cm^{-1}). Red sphere in the center marks the radiation source. (*C*) Distribuion of simulated S_m values of individual cells.

It is assumed that $S_{cs} = S_{cy}$, because microscopy images suggest minimal cytoplasm in NCI-H69 cells (Fig. S2A). S_{cs} and S_{cy} are simulated over MIRDcell V4.14, applying the β average energy spectrum of [^{177}Lu]lutetium on 6 μM radius cells [11], whereby the value $S_{C \leftarrow CS}$ is used for both parameters, because it represents dose deposition inside the nucleus in case that cytoplasm does not exist physically.

For simulating S_m, the cell and radionuclide containing well is virtually established, where the radiation source is centralized and represents the spatially uniformly distributed radionuclides (Fig. S2B). Absorbed dose per activity within a second (S value) is measured by simulating gamma rays emitting from the center, absorbed by the cells (Fig. S2C). Gamma rays were selected to represent beta particles. The script that runs described simulation can be found in the provided GitHub Repository.

Prostate cancer cell lines

Kinetic parameters of the prostate cancer cell lines PC3-PIP (cell radius: 8 µm [12], nucleus radius: 6 µm [13]) and C4-2 (cell radius: 6 µm [12], nucleus radius: 4 µm [13]), which were both seeded in a number of 2'000 cells, were calibrated in the same fashion as for the NCI-H69 cells. Please find the used calibrated parameters and initial conditions for the prostate cancer cell lines in Tab. S3.

Table S8 Used parameters and initial conditions of the prostate cancer cell lines PC3-PIP and C4-2

Parameter or initial condition	PC3-PIP	C4-2
k_{on} [mL nmol^{-1} s^{-1}]	20	20
k_{off} [s^{-1}]	0.2	0.2
k_{int} [s^{-1}]	$3 \cdot 10^{-4}$	$8 \cdot 10^{-4}$
k_{rel} [s^{-1}]	$4 \cdot 10^{-4}$	$2 \cdot 10^{-4}$
R_{cell} [nmol mL^{-1} cell^{-1}]	$1 \cdot 10^{-8}$ [14]	$4 \cdot 10^{-9}$ [15]
S_m [Gy Bq^{-1} s^{-1}]	$2.43 \cdot 10^{-9}$ [13]	$2.43 \cdot 10^{-9}$ [13]
S_{cs} [Gy Bq^{-1} s^{-1}]	$1.07 \cdot 10^{-4}$ [11-13]	$1.84 \cdot 10^{-4}$ [11-13]
S_{cy} [Gy Bq^{-1} s^{-1}]	$1.59 \cdot 10^{-4}$ [11-13]	$3.06 \cdot 10^{-4}$ [11-13]
μ_{gr} [s^{-1}]	$7.7016 \cdot 10^{-6}$ [16]	$1.07526 \cdot 10^{-5}$ [17]
A_m [MBq nmol^{-1}]	40	40
$N_{alive}(0\ h)$	$3.891 \cdot 10^{3}$	$5.064 \cdot 10^{3}$

References

[1] Pommé S, Paepen J, Altzitzoglou T, Van Ammel R, Yeltepe E. Measurement of the 177Lu half-life. Appl Radiat Isot. 2011; https://doi.org/10.1016/j.apradiso.2011.04.021.

[2] Khan MZ, Freshney RI, Murray AM, Merry S, Plumb JA, McNicol AM. Identification and characterisation in vitro of cells with a non-SCLC cell-like phenotype derived from a continuous SCLC cell line. Anticancer Res. 1991; 11(5):1687-95.

[3] Gulston M, Fulford J, Jenner T, de Lara C, O'Neill P. Clustered DNA damage induced by gamma radiation in human fibroblasts (HF19), hamster (V79-4) cells and plasmid DNA is revealed as Fpg and Nth sensitive sites. Nucleic Acids Res. 2002; https://doi.org/10.1093/nar/gkf467.

[4] McMahon SJ, Prise KM. A Mechanistic DNA Repair and Survival Model (Medras): Applications to Intrinsic Radiosensitivity, Relative Biological Effectiveness and Dose-Rate. Front Oncol. 2021; https://doi.org/10.3389/fonc.2021.689112.

[5] Schipler A, Iliakis G. DNA double-strand–break complexity levels and their possible contributions to the probability for error-prone processing and repair pathway choice. Nucleic Acids Res. 2013; https://doi.org/10.1093/nar/gkt556.

[6] McMahon SJ. The linear quadratic model: usage, interpretation and challenges. Phys Med Biol. 2018; https://doi.org/10.1088/1361-6560/aaf26a.

[7] Guidotti G. The composition of biological membranes. Arch Intern Med. 1972; https://doi.org/10.1001/archinte.1972.00320020038003.

[8] Jacobson K, Mouritsen OG, Anderson RG. Lipid rafts: at a crossroad between cell biology and physics. Nat Cell Biol. 2007; https://doi.org/10.1038/ncb0107-7.

[9] Neville DM Jr, Glossmann H. Plasma membrane protein subunit composition. A comparative study by discontinuous electrophoresis in sodium dodecyl sulfate. J Biol Chem. 1971; https://doi.org/10.1016/S0021-9258(18)61793-4.

[10] Taylor JE, Theveniau MA, Bashirzadeh R, Reisine T, Eden PA. Detection of somatostatin receptor subtype 2 (SSTR2) in established tumors and tumor cell lines: evidence for SSTR2 heterogeneity. Peptides. 1994; https://doi.org/10.1016/0196-9781(94)90146-5.

[11] Bolch WE, Eckermann KF, Sgouros G, Thomas SR. MIRD Pamphlet No. 21: A Generalized Schema for Radiopharmaceutical Dosimetry – Standardization of Nomenclature. J Nucl Med. 2009; https://doi.org/10.2967/jnumed.108.056036.

[12] Park S, Ang RR, Duffy SP, Bazov J, Chi KN, Black PC, Ma H. Morphological Differences between Circulating Tumor Cells from Prostate Cancer Patients and Cultured Prostate Cancer Cells. PLoS ONE. 2014, https://doi.org/10.1371/journal.pone.0085264.

[13] Mendelaar PAJ, Kraan J, Van M, Zeune LL, Terstappen LWMM, Oomen-de Hoop E, Martens JWM, Sleijfer S. Defining the dimensions of circulating tumor cells in a large series of breast, prostate, colon, and bladder cancer patients. Mol Oncol. 2021, https://doi.org/10.1002/1878-0261.12802.

[14] Nimmagadda S, Pullambhatla M, Chen Y, Parsana P, Lisok A, Chatterjee S, Mease R, Rowe SP, Lupold S, Pienta KJ, Pomper MG. Low-Level Endogenous PSMA Expression in Nonprostatic Tumor Xenografts Is Sufficient for In Vivo Tumor Targeting and Imaging. J Nucl Med. 2018, https://doi.org/10.2967/jnumed.117.191221.

[15] Lückerath K, Stuparu AD, Wei L, Kim W, Radu CG, Mona CE, Calais J, Rettig M, Reiter RE, Czernin J, Slavik R, Herrmann K, Eiber M, Fendler WP. Detection Threshold and Reproducibility of ^{68}Ga-PSMA11 PET/CT in a Mouse Model of Prostate Cancer. J Nucl Med. 2018, https://doi.org/10.2967/jnumed.118.207704.

[16] Liu J, Huang Y, Zhu D, Dai Y, Liu D, Zhai Y, Liang X, Wu L, Zhao Q. Establishment and characterization of a docetaxel-resistant human prostate cancer cell line. Oncol Lett. 2020, https://doi.org/10.3892/ol.2020.12093.

[17] Castanares MA, Copeland BT, Chowdhury WH, Liu MM, Rodriguez R, Pomper MG, Lupold SE, Foss CA. Characterization of a novel metastatic prostate cancer cell line of LNCaP origin. Prostate. 2016 https://doi.org/10.1002/pros.23115.
